# Isolated reconstruction of the medial patellofemoral ligament with autologous quadriceps tendon

**DOI:** 10.1007/s10195-015-0375-6

**Published:** 2015-09-19

**Authors:** Giovanni Vavalle, Michele Capozzi

**Affiliations:** Department of Orthopaedics, Saint Mary Hospital, De Ferrariis 18/D, 70124 Bari, Italy; Via Martiri della Giustizia, 9, 70016 Noicattaro, Italy

## Abstract

**Background:**

Since the role of the medial patellofemoral ligament (MPFL) as the primary soft-tissue restraint against lateral patellar translation has been recognized, several different reconstruction procedures for the treatment of patellar instability have been proposed over recent years. Many of these techniques require bony procedures and hardware fixation at the patellar and femoral side, leading to complications as described previously in the literature. The purpose of the present study is to describe the technique of isolated MPFL reconstruction using the quadriceps tendon and report the results at a mean follow-up of 38 months. The hypothesis is that this technique, not requiring drilling of bone tunnels on the patellar and femoral side, may be a "simple and safe" mean to manage patellar instability, giving good clinical results with low complication rate in selected patients with normal osseous anatomy.

**Materials and methods:**

Sixteen consecutive patients (9 male, 7 female; mean age 22 years) with chronic patellar instability underwent medial patellofemoral reconstruction with the superficial layer of the quadriceps tendon. All the patients were evaluated preoperatively and postoperatively by physical examination and subjectively with Kujala and Lysholm scores.

**Results:**

The average follow-up was 38 months (range 28–48 months). No recurrent episodes of dislocation or subluxation and no complications occurred. The mean Kujala score increased from 35.8 preoperatively to 88.8 postoperatively and the Lysholm score improved from 43.3 preoperatively to 89.3 postoperatively.

**Conclusions:**

Isolated MPFL reconstruction using an autologous quadriceps tendon and not requiring bone tunnels, may be a safe, simple and effective procedure for the treatment of patellar instability without complications such as patellar fracture as reported by clinical studies using hamstring grafts. For the same reason it may also be indicated in skeletally immature patients.

**Level of evidence:**

Level IV.

## Introduction

Acute patellar dislocation is a common disorder among young active patients with a 50 % recurrence rate [1].

Several bony and soft-tissue etiologic factors can predispose patients to recurrent dislocation or subluxation of the patella. Over the past decade, attention has been directed to the medial patellofemoral ligament (MPFL) as a primary passive soft-tissue restraint to lateral patellar translation at 0–30° of knee flexion, providing approximately 53–67 % of the total medial restraining force [2, 3]. When patellar dislocation occurs, the MPFL is involved in >90 % of cases [4, 5].

When conservative treatment fails, different surgical techniques can be used, including proximal and distal realignment or a combination of both.

Among the proximal procedures, there has been growing interest in the MPFL over recent years.

Many different techniques have been advocated for the reconstruction of the MPFL, but there is no agreement regarding the choice of graft, the type of fixation, the graft positioning, the correct tension and the clinical outcomes. Furthermore, most of the techniques are associated with several complications, such as patellar fracture [6], stiffness [7], hemarthrosis [8], and implant [9] and wound complications [10].

In 2005, Steensen et al. [9] described their original technique using the central one-third of the most superficial layer of the quadriceps tendon, and leaving it attached to the superior pole of the patella. Once the graft is harvested, it is turned 90° medially and fixed to the femoral side with transosseous sutures. Their clinical experience which involved 14 knees in 13 patients showed early promising results, no episodes of recurrent dislocations and no complications.

After 1 year, Noyes et al. [10] presented their surgical technique using the same graft fixed to the medial intermuscular septum with no need for bone tunnels or suture anchors.

Overall, the technique of Steensen et al. seemed simple and less invasive than other procedures previously described in the literature, by not involving bone tunnels and hardware implants.

Therefore, in 2009 we started our clinical experience using a quadriceps graft, focusing on anatomic femoral attachment, which is known to have an important effect on length change pattern of ligaments [11, 12]. Anchor sutures, which are normally used in arthroscopic shoulder rotator cuff tendon surgery, were implanted.

Here, we describe the mid- and long-term results of isolated MPFL reconstruction with an autologous quadriceps graft, in 16 patients affected by recurrent patellar dislocation. The hypothesis was that this technique is a simple and safe way to manage patellar instability, giving good clinical results without any bony procedures or complications, and provides fast recovery.

## Materials and methods

We retrospectively reviewed 16 patients (9 male, 7 female) with recurrent patella dislocation at a mean follow-up of 38 months (range 28–48 months) after index surgery. The average age at the time of surgery was 22 years (range 18–25 years). All patients suffered at least 4 well-documented episodes of unilateral patellar dislocation (mean 8, maximum 20 episodes).

Nine patients practiced sports before the trauma (7 soccer and 2 volleyball); 5 at recreational level and 4 at semi-professional level.

All patients underwent a clinical examination to detect specific signs of patellofemoral pathology and imaging studies (anteroposterior, lateral, standing weight-bearing radiographs of the injured knee and bilateral skyline views at 30° of flexion). Computed tomography (CT) scans were also routinely taken to better evaluate the geometric parameters of the patellofemoral joint.

Exclusion criteria were a tibial tubercle–trochlear grove distance >20 mm, a severe trochlear dysplasia (Dejour grade B, C, D) [13] or a trochlear sulcus angle >145°, patella alta (Insall-Salvati index >1.2) [14], a Q angle >20° in female and >17° in male patients and previous surgeries on the involved knee.

The Lysholm score [15] and the Kujala score [16] were used to evaluate subjective knee function. Furthermore, we assessed the level of satisfaction in relation to pain relief and functional recovery and whether patients had returned or not to their pre-trauma sport and, at which level of competition, at the time of the latest follow-up examination.

Data analysis was performed with paired Students’ *t* test to evaluate differences in preoperative and postoperative subjective outcome scores.

The investigation was performed according to institutionally approved guidelines, having received informed consent from all the patients.

### Surgical technique

**Fig. 1:**
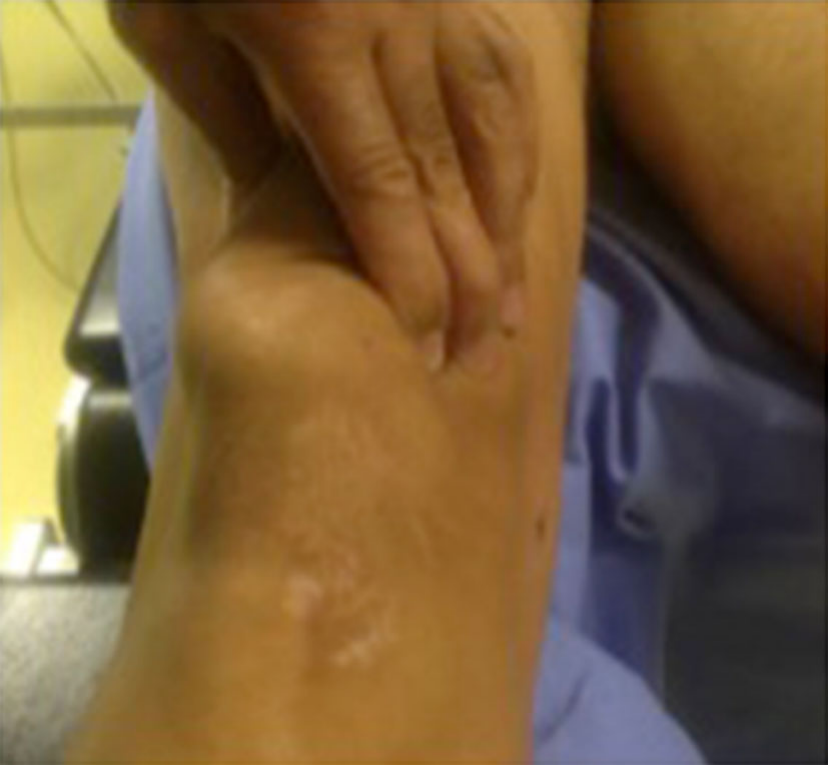
Evaluation under anesthesia before the surgical procedure showing complete patellar lateral dislocation

All procedures were carried out under spinal anesthesia followed by clinical evaluation to confirm the diagnosis of lateral patellar dislocation and to estimate the tightness of the lateral retinaculum (Fig. 1). A manual lateral patellar translation of >50 % of its width from the center of the femoral trochlea was an indirect sign of MPFL incompetence.

With the patient supine and a tourniquet applied to the thigh, a routine diagnostic arthroscopy was carried out to diagnose and treat possible intra-articular lesions, particularly any chondral injuries. Arthroscopic lateral release was performed only if there was excessive tightness of the lateral retinaculum, to avoid the risk of medial patellar translation when the MPFL was reconstructed.

**Fig. 2:**
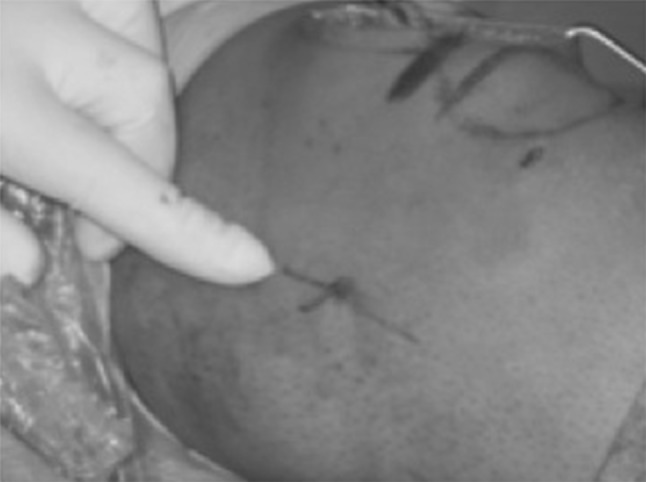
Intraoperative view showing the percutaneous placement of a 1.8-mm K-wire in an isometric femoral insertion

Once the arthroscopy was completed and all surgical instruments were removed, a percutaneous 1.8-mm Kirschner wire (K-wire) was inserted at the isometric femoral point with the use of fluoroscopic guidance as described by Steensen et al. [17] in the beginning of the clinical study and according to the radiographic landmarks defined by Schoettle et al. [18] afterwards (Fig. 2).

A longitudinal incision was performed from the superior pole of the patella, extending proximally for almost 6–7 cm. After identification of the quadriceps tendon, with the knee in flexion, the superficial layer related to the rectus femoris tendon was carefully harvested using a no. 15 scalpel blade, without violating the joint capsule, leaving it attached to the superomedial border of the patellar pole. We tried to harvest the most medial portion of the tendon, leaving 3–4 mm of medial rectus femoris tendon, in order to be closer to the medial border of the patella and to reduce the distance from the side of the femoral attachment. Usually, the harvesting starts 2–3 cm above the patellar pole, where the interval between the superficial and middle layer of the quadriceps tendon is better identified. The native graft width and thickness measured approximately 10–12 and 5 mm, respectively, while the length was estimated by measuring the distance from the superior pole of the patella to the K-wire which was implanted in the medial femoral site, with the knee in a 30° flexion.

Once the appropriate length was reached, the graft was cut proximally and an Ethibond (Ethicon, Somerville, NJ, USA) no. 2 was stitched at the free end with a Krackow continuous suture.

**Fig. 3:**
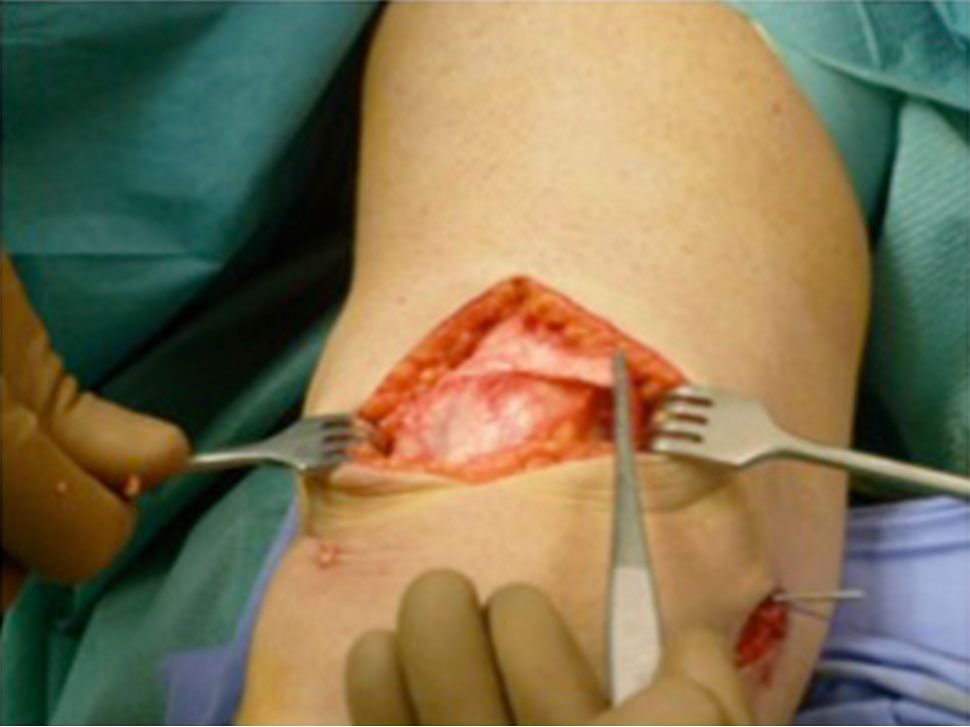
The quadriceps graft is harvested and rotated 90° medially

A careful subperiostal dissection was performed just above its attachment on the patella, paying attention not to amputate the graft, extending further distally on the lateral side so that the graft could be easily turned 90° medially, and lying flush on the anterior surface of the patella (Fig. 3).

To avoid accidental detachment from the patellar insertion, multiple no. 0 nonabsorbable stay stitches were used to fix the graft to the periosteum on the anterior patella.

**Fig. 4:**
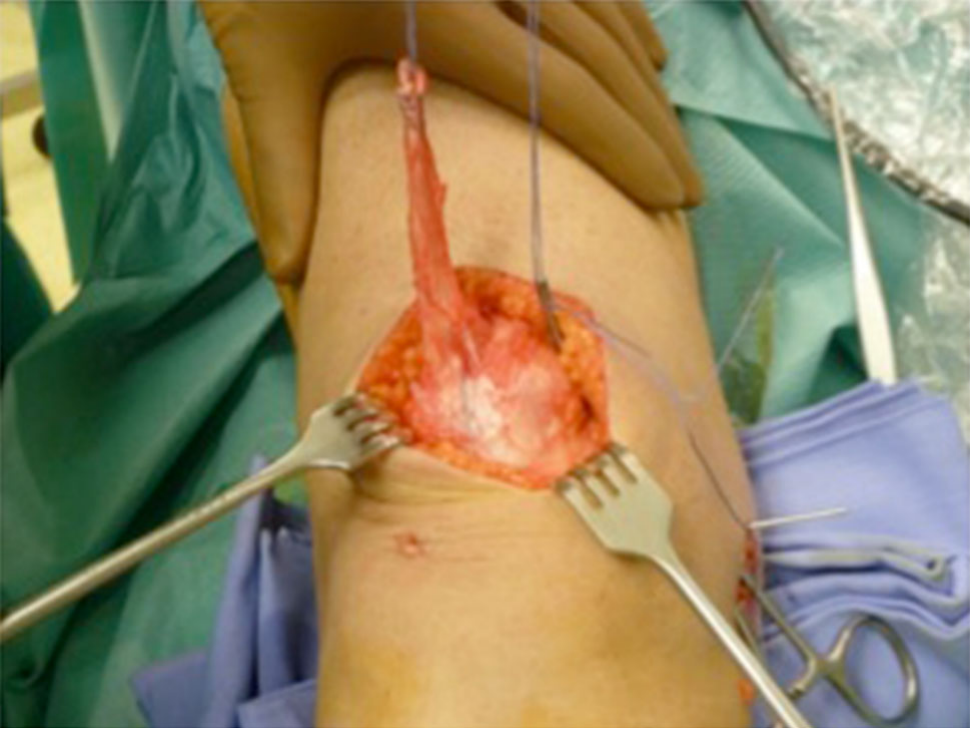
The quadriceps graft is stitched at the free end with no. 2 nonabsorbable sutures and is passed medially through a soft-tissue tunnel with the use of a clamp

A small 2–3 cm longitudinal skin incision was made on the medial femoral epicondyle, involving the implanted K-wire. A blunt dissection was carried out until the bone was exposed. Keeping in mind that the MPFL is located on layer 2 of the medial aspect of the knee, distal to the vastus medialis obliquus (VMO) [17], the graft was passed through a soft tunnel between the deep fascia and the capsule by using a clamp, avoiding penetration of the joint [9] (Fig. 4).

The tourniquet was deflated and hemostasis was performed before the graft was fixed. We believe that an inflated tourniquet can affect patellar tracking due to its binding effect on the extensor mechanism.

**Fig. 5:**
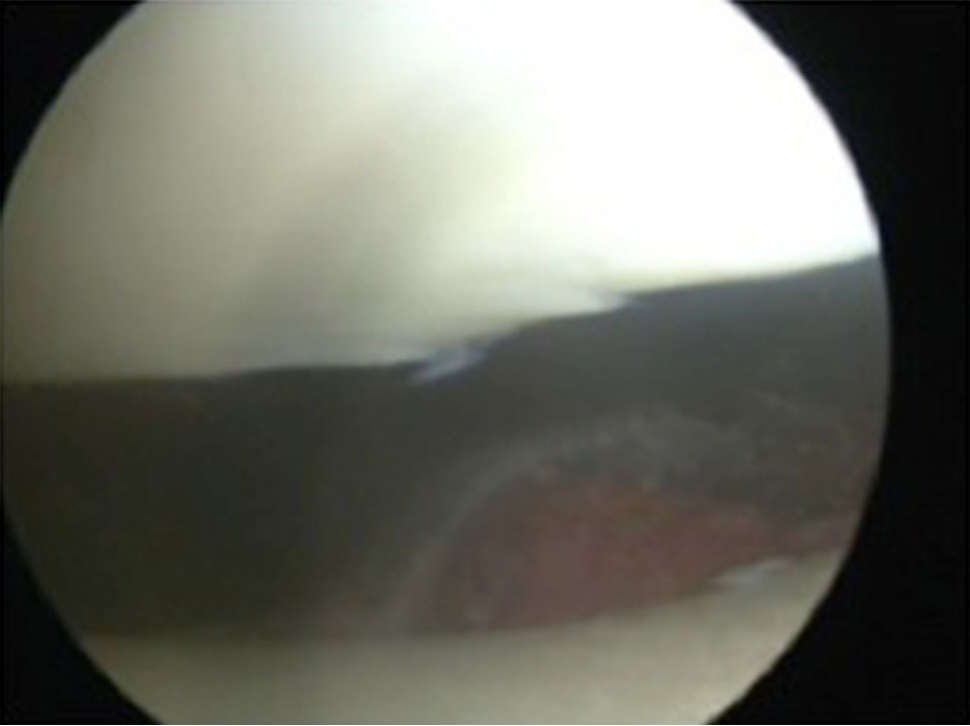
Arthroscopic view showing an optimal patellofemoral congruence

The graft was then tied over the guiding pin and isometry was evaluated by several full flexions and extensions of the knee. It is important not to over-constrain the reconstruction, avoiding increased contact pressures in the patellofemoral joint, and subsequent development of arthrosis. The correct patellar tracking was also assessed dynamically under arthroscopic view (Fig. 5).

**Fig. 6:**
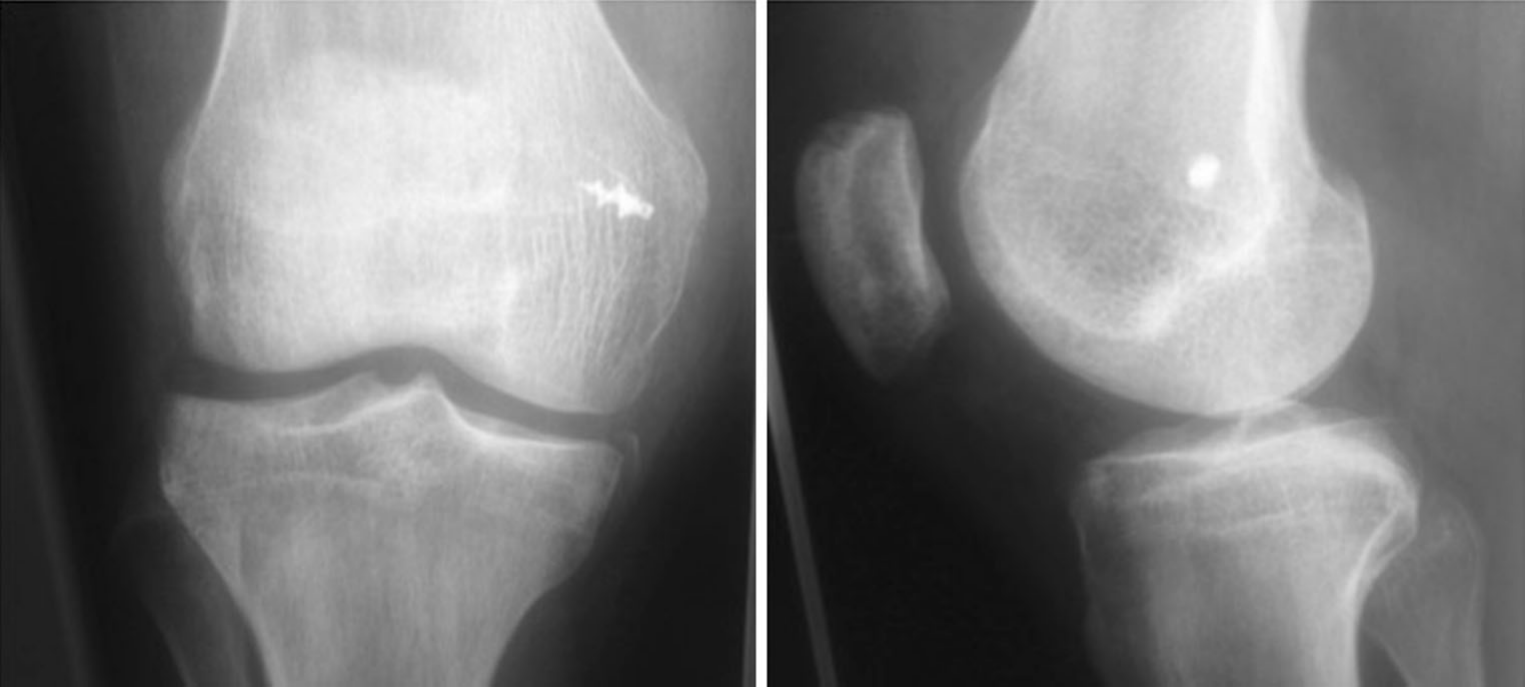
Postoperative radiograph showing the correct insertion of the suture anchor at the isometric femoral point as described by Steensen et al.

The graft was then fixed using a 5.5-mm diameter titanium screw-type anchor (Fastin-RC; Mitek, Westwood, MA, USA) loaded with two strands of no. 2 nonabsorbable Orthocord suture (Ethicon) at approximately 30° knee flexion, where the patella was stabilized into the femoral groove (Fig. 6). In six cases, additional trans-osseus sutures were placed to reinforce the femoral fixation.

The lower border of the VMO was sutured onto the superior limb of the graft with multiple stitches to enhance the reconstruction and improve medial patellar constraint.

The wounds were closed in a standard layered fashion and a hinged brace was worn for 4 weeks after surgery and locked in full extension for the first 3 weeks. Weight bearing was allowed as tolerated with the use of crutches. Quadriceps muscle strengthening was immediately initiated.

A progressive recovery of flexion started the day after surgery, avoiding knee flexion >90° in the first 3 weeks.

## Results

At the final follow-up, no patients reported re-dislocation or subluxation of the patella and no complications occurred postoperatively. All patients regained full range of motion of the knee and no patient showed a positive apprehension test at the most recent follow-up. The endpoint of lateral passive patellar translation was firm in all patients.

During the arthroscopic procedure, Outerbridge [19] stage 2 chondral lesions of the patella were observed in 5 knees (3 in 3 knees and 4 in 2 knees). A free patellar osteochondral fragment was removed from one knee. No degenerative cartilage changes were detected.

The average Kujala score improved significantly from 35.8 ± 5.5 to 88.84 ± 4.3 postoperatively (*p* < 0.001). The mean modified Lysholm score improved significantly from 43.3 ± 6.4 to 89.3 ± 3.1 (*p* < 0.001). The mean improvement in the Kujala score was 53.0 ± 4.3 and the mean improvement in the Lysholm score was 46.0 ± 3.1 (Table 1).

**Table 1:** Functional outcome analysis

	Mean preoperative score	Mean postoperative score	Mean improvement in scores	*p* value
Kujala	35.8 ± 5.5	88.8 ± 4.3	53.0 ± 4.3	<0.001
Lysholm	43.3 ± 6.4	89.3 ± 3.1	46.0 ± 3.1	<0.001

Ten patients rated the surgical procedure as 'very satisfactory' (64 %), 4 as 'satisfied' (21 %) and 2 as 'partially satisfied' (15 %). None were dissatisfied with the surgery.

Partially satisfied results were found in the two patients with Outerbridge stage 3–4 chondral damage.

Five out of 9 patients that practiced sports returned to sports at the same pre-injury level (3 semi-professional and 2 recreational level), 1 reduced their level (from semi-professional to recreational) and 3 retired from sport (all recreational) because they were no longer motivated to continue or for fear of new injuries.

## Discussion

Of the many proximal patellar realignment procedures, MPFL reconstruction has become one of the most frequently used methods since its anatomy and key role in guiding the patella into the femoral groove in the first 30° of knee flexion has been well recognized.

For many years, the MPFL was thought to be an inconstant anatomic structure, present in only 29–88 % of knees [2, 20, 21], and its importance in stabilizing the patella has been underestimated. In fact, being a relatively thin fascial band, the MPFL is sometimes difficult to identify in all knees.

Nowadays, a lesion of the MPFL is considered to be an 'essential lesion', comparable to the Bankart lesion in anterior shoulder instability, without which the patella cannot laterally dislocate [22].

Nomura et al. [23] showed, in a cadaver knee, that by applying a lateral displacing force of 10 N, the patella displaced approximately 6 and 13 mm with an intact and transected MPFL, respectively.

Similar results were found by Hautamaa et al. [24]. In two different studies using a tensile machine on cadaver knees, Conlan et al. [2] and Desio et al. [3] found that MPFL resisted 53–60 % of the force needed to cause a patellar lateral displacement, respectively. They also noted that the role of MPFL in resisting patellar displacement was greater with the knee fully extended, while it became less important for flexions >20°. These findings are supported by anatomic studies [25] on length change patterns of the MPFL, suggesting that the retinacular structures tighten in the extended knee and slacken as it flexes.

Imbrication of the VMO and/or the superficial medial retinaculum has been widely performed over the past years as proximal realignment procedures in patellar instability. Unfortunately, the VMO and superficial medial retinaculum serve only as a minor restraint to lateral displacement, so these procedures do not address the real pathoanatomy of patellar dislocation, which is the MPFL injury.

Multiple techniques have been described recently in the literature for the reconstruction of the MPFL, differing in the type of graft and in the method of fixation to the femur and patella; however, at present there is no consensus as to the best method. Furthermore, no clear clinical superiority of one surgical technique over another is evident.

The hamstrings are the most frequent grafts used in the reconstruction of the MPFL, with different types of fixation. Most of them entail bone tunnels to the patella and to the femoral condyle, sometimes leading to potential complications such as patellar fractures. Many authors [26–28] have already described iatrogenic patellar fractures that occurred because of violation of the anterior patellar cortex during drilling of the bone tunnels.

**Fig. 7:**
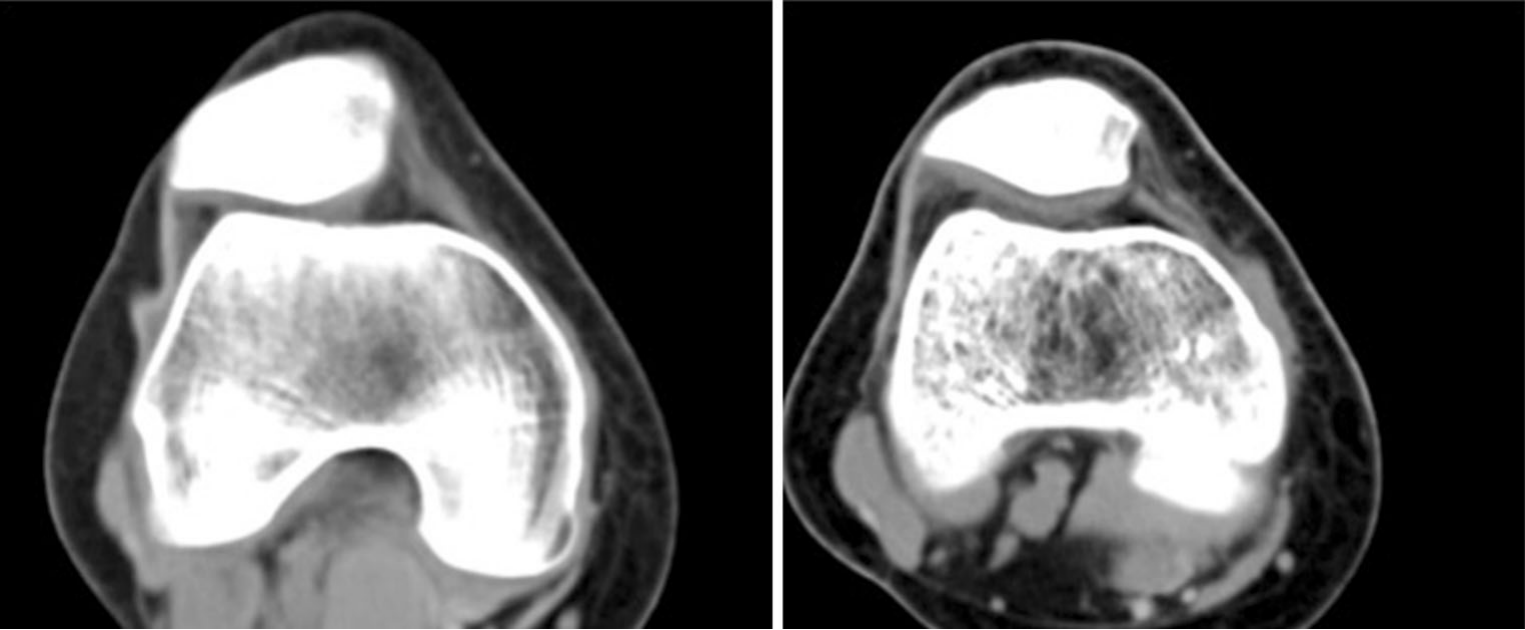
CT scan showing the correction of patellar maltracking after a surgical procedure

Recently, Parikh et al. [29] reported an 16.2 % incidence of complications after MPFL reconstruction in a large series of young patients, using a hamstring graft and performing two bone tunnels on the patella and one on the femur; 47 % of complications were considered to be related to improper technique, including malposition of the femoral tunnel and patellar fractures.

Concerns have also arisen about the relationship between femoral tunnel and distal femoral physis in skeletally immature patients with possible growth disturbance risks.

Furthermore, considering the anatomy of native MPFL as a thin fascial band, hamstring tendons fail to reproduce basic anatomic features. Being thick, cord-like and much stronger, reconstruction with hamstring grafts can lead to medial patellar overload and excessive tensioning of the graft, thus causing graft failure with the risk of patellofemoral arthritis.

Finally, the technique described is also economically advantageous in terms of not requiring the use of implants for patellar fixation.

When we started MPFL reconstructions in 2009, Steensen et al.’s technique [9]), using the most superficial layer of the quadriceps tendon, seemed simpler and less invasive than other procedures described in the literature. This technique did not involve bone tunnels and the need for hardware implants, thus avoiding the complications mentioned above.

Furthermore, by leaving the graft attached to the patellar and then turning it 90° medially, where it is sutured to the patellar retinaculum, the isometric patellar site of the MPFL is easily restored, with no need for bone holes and hardware, thus simplifying the procedure.

The study by Steensen et al. involved 14 knees in 13 patients, with no episodes of recurrent dislocations and no complications.

Therefore, we adopted their technique and tried to improve some critical aspects complying with the most recent biomechanical advancements and anatomic knowledge arising from the international literature.

It is known that femoral insertion of MPFL reconstruction is the most sensitive to reproduce proper ligament isometry, similar to the femoral attachment of the anterior cruciate ligament, so nonanatomic femoral tunnel positioning can lead to unfavorable outcomes [12, 25]. Proximal placement of the femoral attachment might cause tightening of the MPFL reconstruction in flexion with overload of the medial patellar facet. Instead, by placing the femoral origin distally, the MPFL graft tightens in extension, causing non-physiologic patellar motion or stretching the graft.

Therefore, using fluoroscopic guidance, the isometric femoral point is accurately identified according to the radiographic landmarks described by Schoettle et al. [18], and a percutaneous 1.8-mm K-wire is inserted. It is located just anterior to the intersection of the posterior femoral cortical line and Blumensaat line on the lateral radiograph.

Once the graft is harvested and placed under the first layer, it is temporarily tied over the guiding pin and its isometry is evaluated by several full flexions and extensions of the knee, avoiding an over-constrained reconstruction. It is important to verify at the end of the procedure that there is some lateral patellar translation with the knee extended, with a firm lateral endpoint. Furthermore, the correct patellar tracking is also assessed dynamically under arthroscopic view.

To avoid bone tunnels on the femoral side and improve the stability of fixation, the graft is fixed using an 5.5-mm diameter titanium screw-type anchor, normally used in shoulder surgery, loaded with two strands of no. 2 nonabsorbable suture, and occasionally, additional trans-osseus sutures are performed. Thus, this technique can be used regardless of skeletal maturity.

In addition, the lower border of the vastus medialis is sutured distally to the graft, in order to enhance the MPFL, and to give it an active role in the stability of the patella.

In our current study, this surgical technique has proven to be effective. The study included 16 patients with recurrent patella dislocation at a mean follow-up of 38 months who experienced at least 4 well-documented episodes of unilateral patellar dislocation (mean 8, maximum 20 episodes). There were no recurrent dislocations after surgery and no patient showed a positive apprehension test, with 85 % of patients being satisfied/very satisfied (Fig. 7). No complications occurred postoperatively. Our results are similar to previous studies [30–32].

The limited number of patients involved in the current study is related to highly careful preoperative selection, performing isolated reconstruction of MPFL only in cases without any predisposing factors such as abnormal Q angle, trochlear dysplasia, patella alta and malalignment of the lower extremity.

Although this is a small study, there are several interesting observations that can be made. First, all procedures were performed by one surgeon (GV). Second, the average follow-up rate was 38 months, i.e., a period long enough to evaluate the effectiveness of a surgical procedure in the treatment of patellar instability.

Some limitations to this study must also be considered. The postoperative evaluations were not blinded and there was no control group. In addition, being retrospective in nature, our study has limitations similar to other retrospective studies.

In conclusion, MPFL reconstruction using a quadriceps tendon graft is a relatively simple procedure which eliminates the need for bone tunnels. It is safe (given that we observed no complications) and effective with no cases of dislocation or subluxation and with a patient satisfaction rate >80 %. Correct indications and overall careful preoperative evaluation to rule out any bony abnormalities, are key to successful long-term outcomes. As the technique does not require bone tunnels to be drilled, it may be indicated in skeletally immature patients [33].
